# Identification and Analysis of Hub Genes and Immune Cells Associated with the Formation of Acute Aortic Dissection

**DOI:** 10.1155/2023/8072369

**Published:** 2023-02-08

**Authors:** Aifang Zhong, Yuzhong Cai, Yang Zhou, Ning Ding, Guifang Yang, Xiangping Chai

**Affiliations:** ^1^Department of Emergency Medicine, The Second Xiangya Hospital, Central South University, Changsha, Hunan, China; ^2^Trauma Center, The Second Xiangya Hospital, Central South University, Changsha, Hunan, China

## Abstract

**Background:**

Acute type A aortic dissection (AAD) is a catastrophic disease with high mortality, but the pathogenesis has not been fully elucidated. This study is aimed at identifying hub genes and immune cells associated with the pathogenesis of AAD.

**Methods:**

The datasets were downloaded from Gene Expression Omnibus (GEO). Gene Set Enrichment Analysis (GSEA), gene set variation analysis (GSVA), and differential analysis were performed. The differentially expressed genes (DEGs) were intersected with specific genes collected from MSigDB. The gene function and pathway enrichment analysis were also performed on intersecting genes. The key modules were selected by weighted gene coexpression network analysis (WGCNA). Hub genes were identified by least absolute shrinkage and selection operator (LASSO) analysis and were verified in the metadataset. The immune cell infiltration was analyzed by CIBERSORT, and the relationship between hub genes and immune cells was performed by Pearson's correlation analysis. The single-cell RNA sequencing (scRNA-seq) dataset was used to verify the differences in DNA damage and repair signaling pathways and hub genes in different cell types.

**Results:**

The results of GSEA and GSVA indicated that DNA damage and repair processes were activated in the occurrence of AAD. The gene function and pathway enrichment analysis on differentially expressed DNA damage- and repair-related genes showed that these genes were mainly involved in the regulation of the cell cycle process, cellular response to DNA damage stimulus, response to wounding, p53 signaling pathway, and cellular senescence. Three key modules were identified by WGCNA. Five genes were screened as hub genes, including CDK2, EIF4A1, GLRX, NNMT, and SLCO2A1. Naive B cells and Gamma delta T cells (*γδ* T cells) were decreased in AAD, but monocytes and M0 macrophages were increased. scRNA-seq analysis included that DNA damage and repair processes were activated in smooth muscle cells (SMCs), tissue stem cells, and monocytes in the aortic wall of patients with AAD.

**Conclusions:**

Our results suggested that DNA damage- and repair-related genes may be involved in the occurrence of AAD by regulating many biological processes. The hub genes and immune cells reported in this study also increase the understanding of AAD.

## 1. Introduction

Aortic dissection (AD) is an emergency in which the intima of the aorta is partially ruptured due to a variety of causes. The blood then enters the media or outer layers of the aorta, stratifying the vessel wall to form a “dual-lumen aorta” [1]. According to the different parts of the aorta involved, Stanford classification divides AD into type A AD (AAD) and type B AD (BAD). AAD has an acute onset and rapid progression, and the in-hospital mortality rate is higher than that of BAD (22% vs. 14%) [2]. Although aggressive surgical intervention for acute AAD improves the survival rate, patients may develop many postoperative complications so that their quality of life decreases [3]. There is still a lack of effective methods to intervene in the occurrence and development of AAD because the pathogenesis of AAD has not yet been fully explained. Therefore, it is necessary to identify new diagnostic biomarkers and find the potential molecular mechanisms of AAD.

The cells are constantly responding to endogenous processes (such as oxidative stress and DNA replication stress) or exogenous exposures (such as UV radiation and chemotherapy drugs) that cause damage to DNA [4–6]. Whenever DNA damage occurs in cells, the body will spontaneously sense and repair the damage; this response is called DNA damage response (DDR) [6, 7]. However, when DNA damage persists in cells or the damage is not repaired normally, it may lead to gene instability, autophagy, senescence, and finally cell death [5, 6, 8]. Studies have revealed that DNA damage and enzyme system repair defects are associated with the pathogenesis of pulmonary hypertension, systemic lupus erythematosus, Alzheimer's disease, atherosclerosis, aortic disease, and many other diseases [7, 9–12]. Research indicated that evidence of unrepaired DNA double-strand structure breaks in 90% of aortic disease samples, and 28.7% of smooth muscle cells (SMCs) had foci of DNA damage [11]. Studies also observed a large amount of DNA damage in the smooth muscle of thoracic aortic aneurysm (AA) tissues [13, 14]. The nuclear and mitochondrial DNA damage in SMCs of AA and AD tissues and subsequent DNA leakage into the cytoplasm activates the STING signaling, which induces cell death through apoptosis and necrosis [15]. Although studies have confirmed that DNA damage is involved in the occurrence of AD, DNA damage and repair are complex regulatory networks, and few studies systematically explore the relationship between the genes related to DNA damage and repair and AAD. Inflammation is an important factor leading to the occurrence and progression of AD [16–18]. A variety of immune cells can participate in the occurrence of AAD by switching to a proinflammatory phenotype and releasing inflammatory factors [18–20]. A previous study found that DDR-related proteins were involved in inflammatory responses [7]. But in AAD disease, the relationship between genes related to DNA damage and repair and inflammatory and immune cells has not been elucidated.

The development of gene sequencing technology has made it a trend to explore the mechanisms and biomarkers of facilitated the study of the pathogenesis or progression of AAD at the molecular level. A study performed sequencing on ascending aortic tissue from patients with AAD, and controls indicated that activated inflammation, SMC degeneration, and cell death are the main pathological changes in AAD. Meanwhile, they identified ten potential hub genes regulated to autophagy [21]. Single-cell RNA sequencing (scRNA-seq) technology can observe changes that occur in different cell types [22]. Xu et al. identified 15 cell clusters and nine cell types in AD mouse tissues by scRNA-seq, and they found that the type and relative number of SMCs changed significantly in AD model mouse tissues [23].

Therefore, this study explored the relationship between DNA damage and repair genes with AAD by downloading sequencing datasets from the public database. Weighted gene coexpression network analysis (WGCNA) was performed to identify the key modules closely related to AAD; hub genes identified by the least absolute shrinkage and selection operator (LASSO) regression analysis. The relationship between hub genes and immune cells was explored by Pearson's correlation analysis. Particularly, this study demonstrates the changes in DNA damage and repair response from two levels of the bulk RNA sequencing and scRNA-seq datasets, which is distinct from other studies.

## 2. Materials and Methods

### 2.1. Data Processing

The four datasets (GSE15343/GSE52093/GSE98770/GSE213740) [21, 24, 25] were downloaded from the public database Gene Expression Omnibus (GEO). The high throughput sequencing dataset GSE153434 (GPL20795) contains gene expression information in ten ascending aortic tissues from the patients with AAD and ten normal aortic tissues. The microarray dataset GSE52093 (GPL10558) includes aortic tissues from the seven patients with AAD and five normal aortic tissues, and the GSE98770 (GPL14550) includes six AAD and five aortic tissues without AAD. The dataset GSE153434 was differentially analyzed using the “DESeq2” package [26] of the R software (version 4.1.2) [27]. Similarly, background correction and normalization were also performed using the “DESeq2” package. The counts' value of GSE153434 was then converted to TPM for immune cell infiltration analysis and Gene Set Enrichment Analysis (GSEA). The “limma” package [28] was used to read the raw data of datasets GSE52093 and GSE98770. The two datasets were merged into a metadataset by the “sva” package, which was used as a validation dataset. GSE213740 is a scRNA-seq dataset including ascending aortic wall tissue of six AAD patients and three donors.

### 2.2. Gene Set Enrichment Analysis and DNA Damage- and Repair-Related Gene Collection

Gene enrichment analysis was performed in GSEA software [29, 30]. Combined with the results of the GSEA, gene sets that are related to DNA damage and DNA repair were downloaded from the MSigDB database [30] (GSEA|MSigDB (http://gsea-msigdb.org)). After screening and deduplication, 1089 genes were included in this analysis (Supplementary Table 1). The overall trend of 1089 genes in AAD and controls, which represented DNA damage and repair response, was assessed by the gene set variation analysis (GSVA) algorithm.

### 2.3. Identification of Differentially Expressed DNA Damage- and Repair-Related Genes

The genes with ∣log_2_ FC | >1 and *P* < 0.05 were screened as differentially expressed genes (DEGs) in GSE153434, and the “ggplot2” package [31] was used to draw the volcano plot of DEGs. The DEGs and DNA damage- and repair-related genes were uploaded to the online website Draw Venn Diagram (Draw Venn Diagram (ugent.be)) for the intersection. The top 25 upregulated and downregulated genes of the log_2_ FC value rank in the intersection genes were screened, respectively, and visualized with a heatmap.

### 2.4. Gene Functional and Pathway Enrichment Analyses

The differentially expressed DNA damage- and repair-related genes were uploaded to the online website Metascape [32] for functional enrichment, and the results were downloaded. Disease Ontology (DO) enrichment analysis was performed with the enrichDO function of R software [33, 34], and Kyoto Encyclopedia of Genes and Genomes (KEGG) pathway analysis was also performed in R and visualized with a bubble diagram. *P* < 0.05 was considered meaningful.

### 2.5. Construction of Protein-Protein Interaction (PPI) Network

A PPI network was constructed by the STRING website [35] and visualized in Cytoscape (version 3.9.0) software.

### 2.6. Weighted Gene Coexpression Network Analysis and Identification of Key Modules

WGCNA was conducted based on 1089 DNA damage- and repair-related genes to explore the modules closely related to AAD. Samples in GSE153434 were clustered to remove outlier samples. The pickSoftThreshold function was used to calculate the soft threshold, and the optimal soft threshold was chosen to build a scale-free network (Supplementary Figure 1). A stepwise approach was used to construct gene networks and identify modules with >40 genes, and similar modules were subsequently merged. The correlation between each module and the clinical trait was calculated by the Spearman method, which is a built-in function in the “WGCNA” package. *P* < 0.05 was considered significant.

### 2.7. Identification and Validation of Hub Genes

The intersecting genes were obtained from the key modules' genes and DEGs. LASSO regression analysis of the “glmnet” package in R was used to screen for hub genes associated with AAD in intersecting genes. Subsequently, receiver operating characteristic (ROC) curves and the area under the curve (AUC) values were used to evaluate the predictive value of hub genes. The expression of hub genes was validated in the metadataset and visualized by a boxplot.

### 2.8. Analysis of Immune Cell Infiltration

CIBERSORT [36] estimates the abundance of immune cells based on the gene expression data by the deconvolution method. The data (LM22.txt) on the gene expression characteristics of 22 kinds of immune cells were downloaded from the official website of CIBERSORT (Supplementary Table 2). Immune cell infiltration analysis was performed on 20 samples in GSE153434 in R software as well. The “ggplot2” package was used to visualize the immune cell abundance results of the samples. The cells with a content of 0 in each sample were filtered out. The Wilcoxon test was used to analyze the differences in the immune cell content between AAD and controls. Pearson's correlation analysis was performed on the hub genes and immune cells, and the packages of “ggpubr” [37] and “ggplot2” were used to draw the correlation scatterplots.

### 2.9. scRNA-seq Analysis

The “Seurat” package [38] was used for reading data and analysis, and the “SingleR” package [39] was used to annotate cell types. The cells with the number of genes (nFeature RNA) ≤ 200 or ≥2500 were filtered out. Dimensionality reduction and clustering were performed, and 3000 hypervariable genes were selected for the subsequent analysis. The first 15 dimensions were chosen for follow-up analysis according to JackStrawPlot and ElbowPlot. The AddModuleScore function in “Seurat” was to score 1089 genes related to DNA damage and repair. Cellular clusters and gene expression profiles were visualized by DimPlot and VlnPlot functions, respectively.

## 3. Results

### 3.1. Data Processing

**Figure 1:**
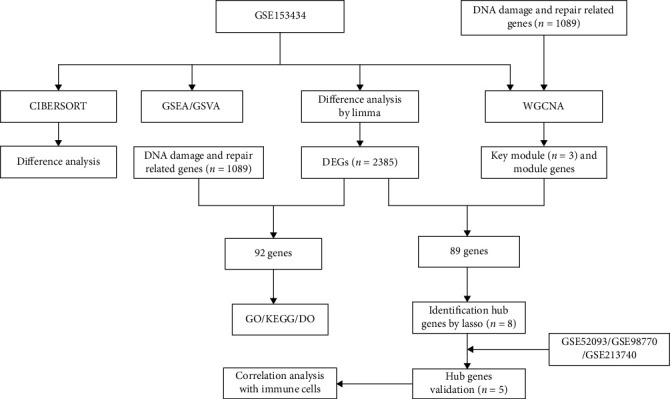
The framework of the study.

Principal component analysis (PCA) before and after merging the GSE52093 and GSE98770 is presented in Supplementary Figure 2, showing that the batch effect between the datasets was eliminated. The framework of this study is shown in Figure 1.

### 3.2. Gene Set Enrichment Analysis and Gene Set Variation Analysis

**Figure 2:**
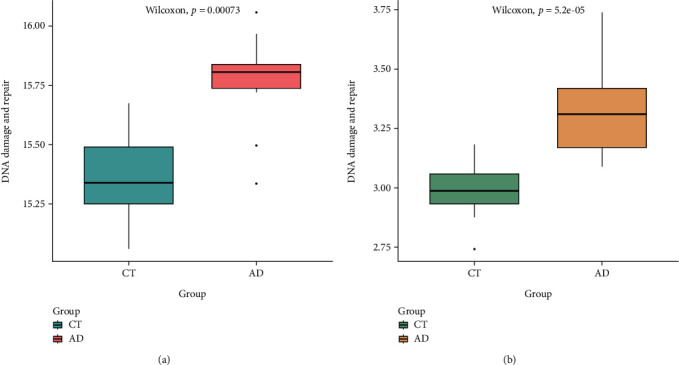
The results of gene set variation analysis (GSVA) in GSE153434 and metadataset. (a) The boxplot shows that DNA damage and repair were an upregulation in the AAD group in GSE153434. (b) DNA damage and repair were an upregulation in the AAD group in the metadataset.

Through GSEA of the gene expression profile on GSE153434, four biological processes, including nucleotide excision repair, base excision repair, DNA repair, and G2M checkpoint, were significantly enriched in the AAD group (Supplementary Figure 3), which suggests that DNA damage and repair response may involve in the pathogenesis of AAD. The result of GSVA shows that DNA damage and repair processes were activated in the AAD group, and a consistent trend was also observed in the metadataset (Figure 2).

### 3.3. Identification of Differentially Expressed DNA Damage- and Repair-Related Genes

**Figure 3:**
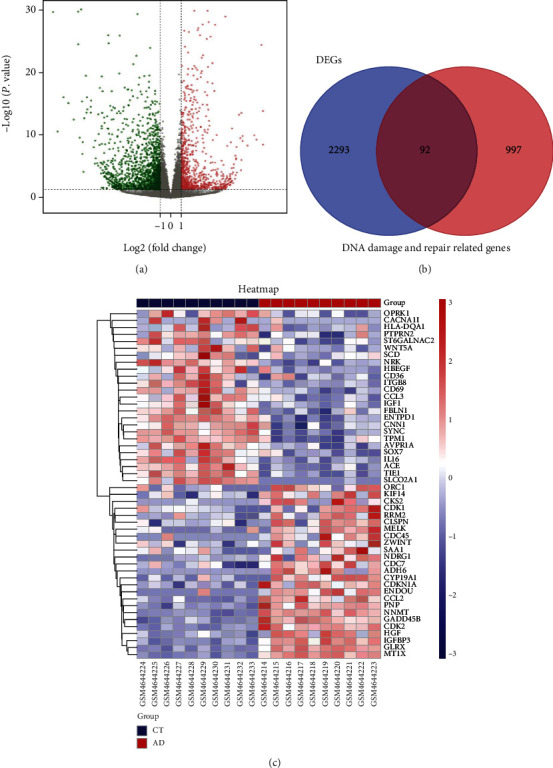
Volcano plots of DEGs and heatmap of differentially expressed DNA damage- and DNA repair-related genes. (a) Volcano plot of DEGs in GSE153434. The red dots represent upregulated genes, the green dots represent downregulated genes, and the grey dots represent genes without significant differences. (b) The Venn diagram shows the intersection of DEGs and DNA damage- and repair-related genes. (c) The heatmap shows the top 25 upregulated and downregulated genes of differentially expressed DNA damage- and repair-related genes.

A total of the 2385 DEGs were screened from the dataset GSE153434, including 889 upregulated and 1496 downregulated (Figure 3(a)). We collected 1089 DNA damage- and repair-related genes from MSigDB. Finally, 92 intersecting genes (Supplementary Table 3) were obtained in this study, including 50 upregulated genes and 42 downregulated genes, which were regarded as differentially expressed DNA damage- and repair-related genes (Figure 3(b)). The top 25 upregulated and downregulated genes of the log2FC value rank were visualized with a heatmap (Figure 3(c)).

### 3.4. Gene Functional and Pathway Enrichment Analyses

**Figure 4:**
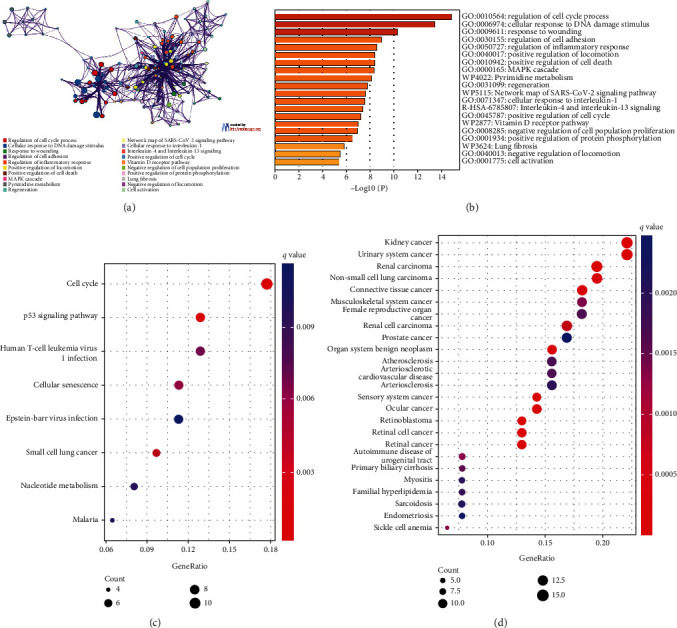
Gene functional enrichment analysis. (a, b) The results of enrichment analysis on the Metascape website. (c) Bubble diagram of the KEGG enrichment analysis. (d) Bubble diagram of the DO enrichment analysis.

The results of enrichment analysis on the Metascape website indicated that differentially expressed DNA damage- and repair-related genes were mainly enriched in the regulation of cell cycle process, cellular response to DNA damage stimulus, response to wounding, involved in the regulation of cell adhesion, regulation of inflammatory response, positive regulation of locomotion, and cell death biological processes (Figures 4(a) and 4(b)). KEGG enrichment analysis shows that these genes were mainly enriched in the cell cycle, p53 signaling pathway, and cellular senescence (Figure 4(c)). The results of DO enrichment analysis showed that genes related to DNA damage and repair involved in the formation of AAD were also involved in the processes of atherosclerosis, arteriosclerotic cardiovascular disease, familial hyperlipidemia, and arteriosclerosis (Figure 4(d)).

### 3.5. PPI Network Construction

The 92 DNA damage- and repair-related genes were included in the PPI network, and the minimum interaction score of the network was 0.4. The network was modified by Cytoscape software (Supplementary Figure 4).

### 3.6. Weighted Gene Coexpression Network Analysis and Identification of Key Modules

**Figure 5:**
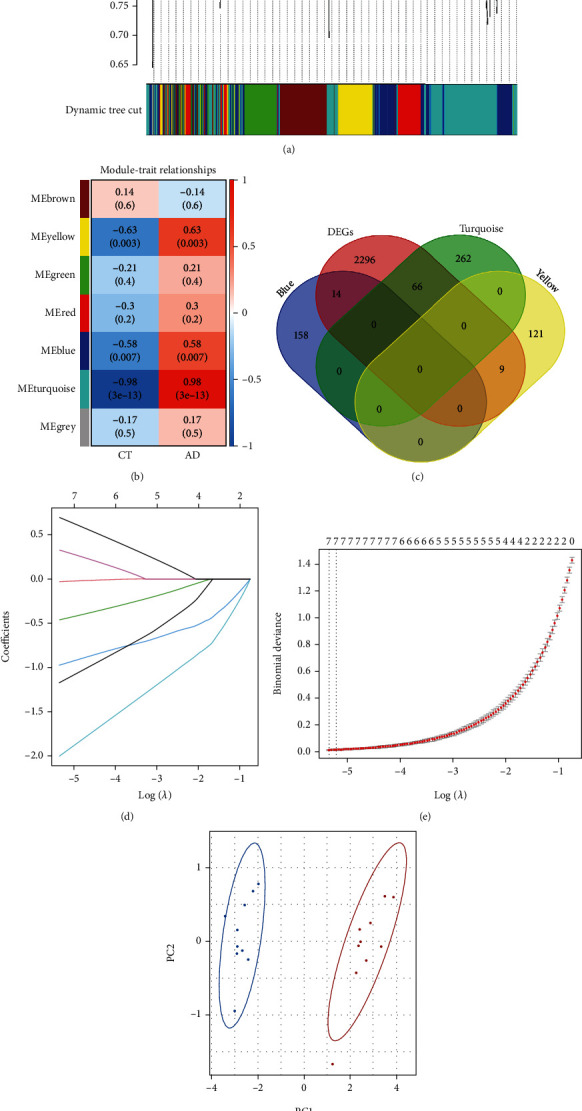
Weighted gene coexpression network analysis (WGCNA) and least absolute shrinkage and selection operator (LASSO) analysis. (a) The cluster dendrogram of genes in GSE153434. (b) The relationships between modules and the trait. Each cell contains the corresponding correlation and *P*. (c) A Venn diagram shows the intersecting genes obtained from the DEGs and three key module genes. (d, e) The LASSO analysis identified eight hub genes associated with AAD. (f) PCA of between the samples of AAD and controls based on eight hub genes.

To reveal the differences between AAD and controls, WGCNA was performed based on 1089 DNA damage- and repair-related genes (Figure 5(a)). The correlation analysis revealed that three functional modules (blue, yellow, and turquoise) were closely related to AAD (*P* < 0.05), as shown in Figure 5(b).

### 3.7. Identification and Validation of Hub Genes

**Figure 6:**
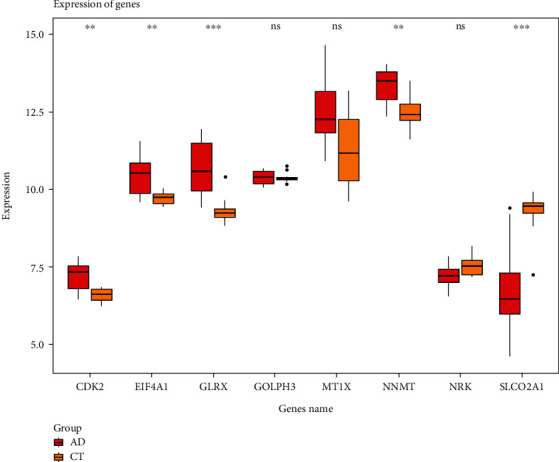
Validation of the hub genes in metadataset. ^∗∗^*P* < 0.01 and ^∗∗∗^*P* < 0.001. ns indicates nonsignificant.

Genes in the blue, yellow, and turquoise modules were intersected with DEGs, and 89 intersecting genes were obtained (Figure 5(c)). LASSO regression analysis was used to filter the intersecting genes (Figures 5(d) and 5(e)), and eight hub genes associated with the occurrence of AAD were eventually identified, namely, cyclin-dependent kinase 2 (CDK2), EIF4A1, glutaredoxin (GLRX), Golgi phosphoprotein 3 (GOLPH3), metallothionein 1X (MT1X), nicotinamide N-methyltransferase (NNMT), Nik-related kinase (NRK), and solute carrier organic anion transporter family member 2A1 (SLCO2A1). ROC curves of hub genes are shown in Supplementary Figure 5. The PCA based on eight hub genes could well distinguish AAD samples from control samples (Figure 5(f)). The expression of hub genes was verified in the metadataset (Figure 6), and the results show that genes of GOLPH3, MT1X, and NRK had no difference between the AAD group and the control group (*P* > 0.05).

### 3.8. Analysis of Immune Cell Infiltration

**Figure 7:**
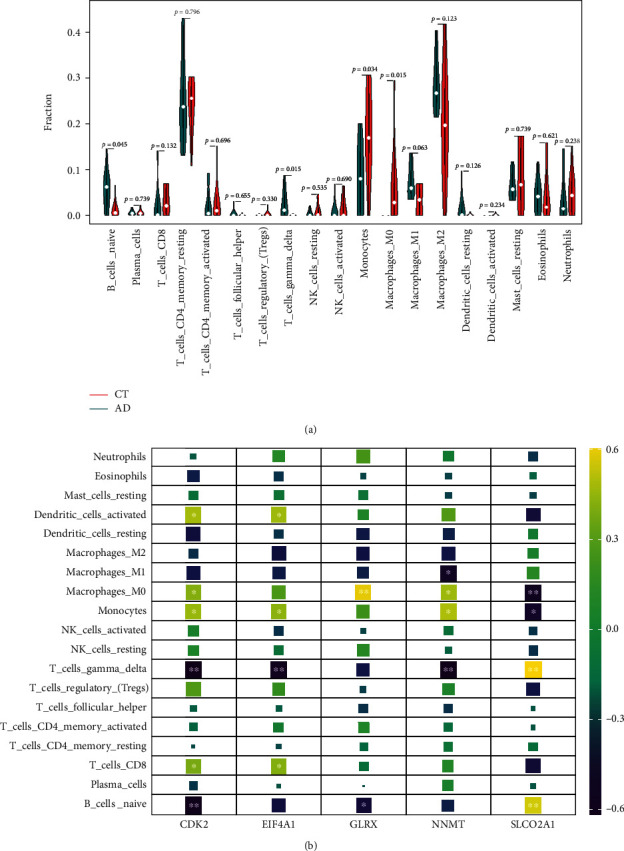
Composition of infiltrating immune cells in aortic tissues. (a) The violin plot of infiltrating immune cells between AAD samples and control samples. (b) Correlation analysis between infiltrating immune cells and hub genes. ^∗^*P* < 0.05 and ^∗∗^*P* < 0.01.

The infiltration abundance of 22 types of immune cells in each sample is shown in Supplementary Figure 6A, and three types of immune cells with 0 in the expression of each sample amount were deleted, including memory B cells, naive CD4+ T cells, and activated mast cells. The correlation analysis on immune cells revealed that monocytes were negatively correlated with M2 macrophages and Gamma delta T cells (*γδ* T cells), naive B cells were positively correlated with *γδ* T cells, and M0 macrophages were negatively correlated with M2 macrophages (Supplementary Figure 6B). There were four different abundances of immune cells between AD tissue and control tissue. Both naive B cells (*P* = 0.045) and *γδ* T cells (*P* = 0.015) were decreased in AAD tissue, but monocytes (*P* = 0.034) and M0 macrophages (*P* = 0.015) were increased in AAD tissue (Figure 7(a)). The correlations between hub genes and immune cells are shown in Figure 7(b). The expression of CDK2 was negatively correlated with the abundance of *γδ* T cells and naive B cells, while it was positively correlated with the abundance of M0 macrophages, monocytes, CD8+ T cells, and activated dendritic cells. The expression of EIF4A1 was negatively correlated with the abundance of *γδ* T cells, but it was positively correlated with the abundance of monocytes, CD8+ T cells, and activated dendritic cells. The expression of gene GLRX was positively correlated with M0 macrophages, while it was negatively correlated with naive B cells. The expression of NNMT was negatively correlated with the abundance of M1 macrophages and *γδ* T cells, and it was positively correlated with the abundance of M0 macrophages and monocytes. The expression of SLCO2A1 was positively correlated with the abundance of *γδ* T cells and naive B cells, and it was negatively correlated with the abundance of M0 macrophages and monocytes.

### 3.9. Single-Cell RNA Sequencing Analysis

**Figure 8:**
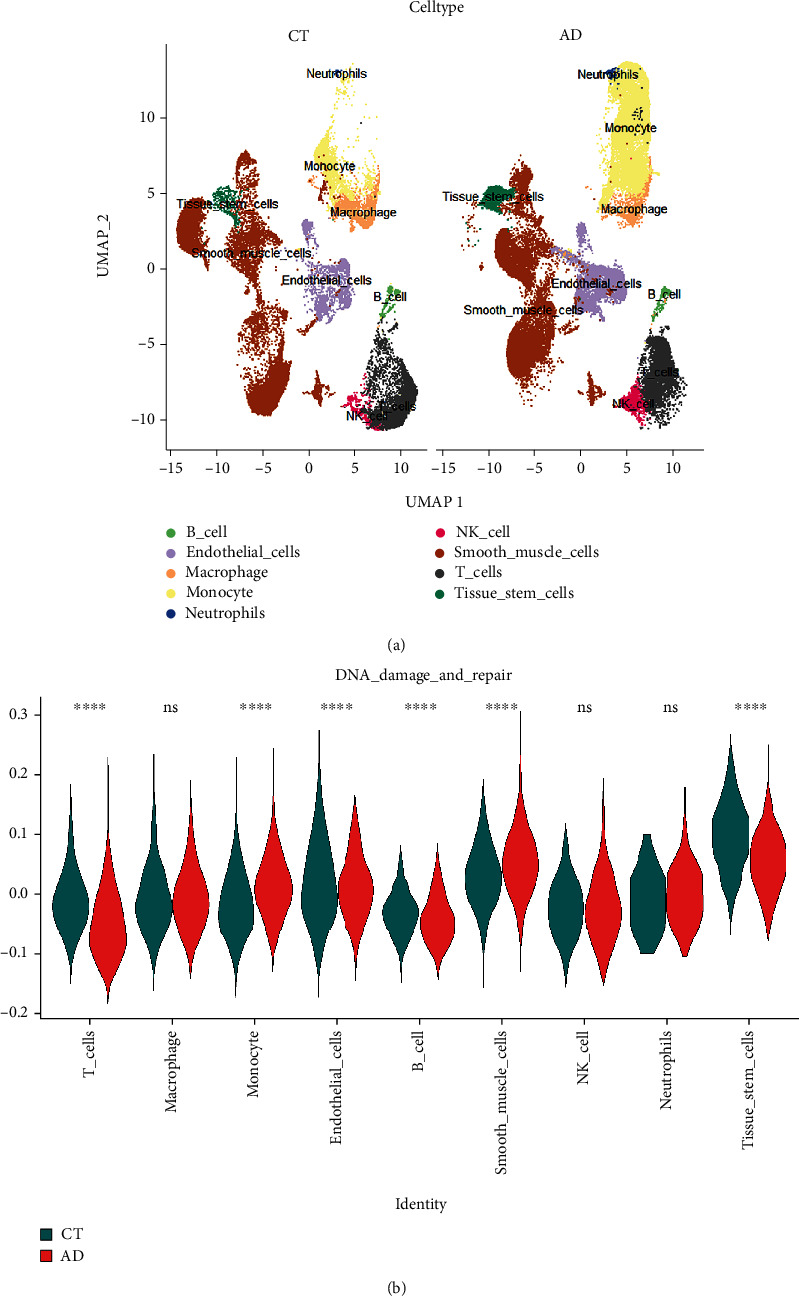
Single-cell RNA sequencing analysis. (a) The nine major cell types were identified. (b) The violin plot shows the difference in the score of DNA damage- and repair-related genes in various cells between AAD patients and normal donors. ^∗∗∗∗^*P* < 0.0001. ns indicates nonsignificant.

**Figure 9:**
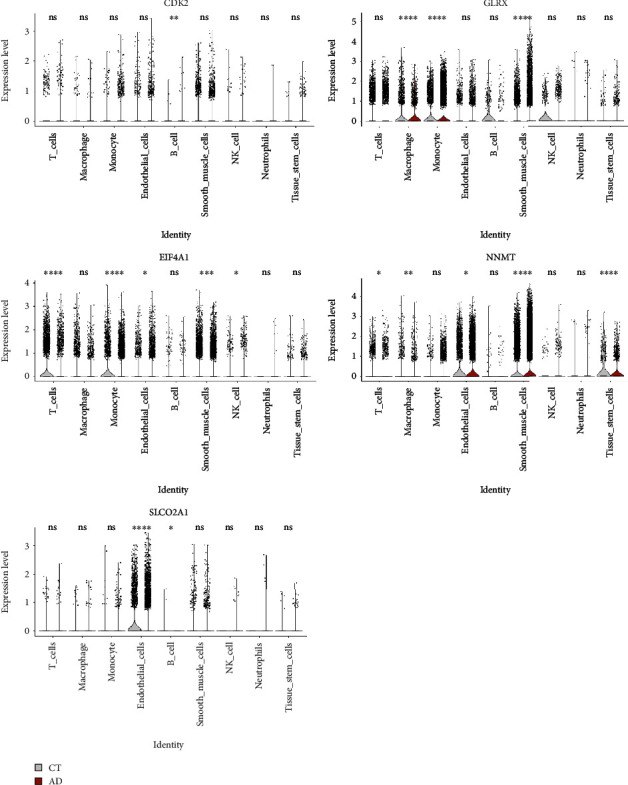
The violin plots show expression levels of hub genes in 9 cell types between AAD patients and normal donors. ^∗^*P* < 0.05, ^∗∗^*P* < 0.01, ^∗∗∗^*P* < 0.001, and ^∗∗∗∗^*P* < 0.0001. ns indicates nonsignificant.

The violin plot showed RNA characteristic number (nFeature RNA) and absolute UMI count (nCount RNA) after low-quality cells were removed (Supplementary Figure 7A). We identified 27 different cell clusters and nine cell types in this study (Supplementary Figure 7B and Figure 8(a)). The nine cell types included three nonimmune cells (endothelial cells, tissue stem cells, and SMCs) and six immune cells (B cells, monocyte, natural killer T cell, macrophage, neutrophils, and T cells) (Figure 8(a)). The results show that DNA damage and repair responses were significantly activated in a variety of cells in the aortic wall of patients with AAD through AddModuleScore function analysis, such as SMCs, endothelial cells, and monocyte, which was consistent with previous bulk RNA sequencing results (Figure 8(b)). Interestingly, DNA damage and repair responses were inhibited in T cells, B cells, and tissue stem cells of aortic wall tissue. Finally, we calculated the expression of five hub genes in nine cell types between AAD and normal (Figure 9). Through analysis, we found that the CDK2 gene was mainly expressed in SMCs and upregulated in SMCs and B cells. EIF4A1 and GLRX were widely expressed in SMCs, T cells, and monocytes, which were upregulated in SMCs and monocytes, but EIF4A1 downregulated in T cells. NNMT gene was mainly expressed on SMCs and endothelial cells, which was upregulated in SMCs and endothelial cells; SLCO2A1 was mainly expressed on endothelial cells and was upregulated, which differed from the bulk RNA sequencing data.

## 4. Discussion

At present, some studies have focused on the existence of DNA damage in aortic SMCs in patients with AD and aneurysms and confirmed that DNA damage could lead to SMC senescence and death [15, 40, 41]. Through GSEA, the four pathways activated in AAD patients supported the involvement of DNA damage and repair processes in the occurrence of AAD. For further exploration of the function of genes related to DNA damage and repair in AD, we collected 1089 DNA damage and repair-related genes in this study. The results of GSVA were strong evidence that DNA damage and repair processes were activated in the occurrence of AD. A total of 92 genes related to DNA damage and repair were obtained, including 50 upregulated genes and 42 downregulated genes. Gene enrichment analysis indicated that these genes were mainly involved in the regulation of cell cycle process, cellular response to DNA damage stimulus, and response to wounding. KEGG analysis revealed that the 92 genes were mainly enriched in the cell cycle, p53 signaling pathway, and cellular senescence. The three key modules closely related to AD were identified through WGCNA. The five hub genes were verified, including CDK2, EIF4A1, GLRX, NNMT, and SLCO2A1. The immune cell infiltration analysis indicated that naive B and *γδ*T cells were decreased in AAD, but monocytes and M0 macrophages were increased. The analysis of correlation exposed that five hub genes have existed in correlation with immune cells. At last, scRNA-seq analysis showed that DNA damage and repair processes were significantly activated in SMCs, endothelial cells, and monocyte in the aortic wall of patients with AD. The hub genes were all highly expressed on SMCs, except SLCO2A1 mainly expressed on endothelial cells.

Hypertension, dyslipidemia, smoking, atherosclerosis, and male gender are important risk factors for AD [42–45]. Dyslipidemia is a common cause of the development of atherosclerosis. When the endothelial function is compromised, low-density lipoprotein (LDL) particles may deposit in the arterial wall to form aggregates with intimin. These aggregates enter SMCs via receptors of the LDL receptor-related protein (LRP) superfamily. The accumulation of lipids in cells promotes arterial plaque formation. As atherosclerosis progresses, SMCs produce extracellular matrix (ECM) molecules that contribute to intimal thickening, resulting in fibrosis and calcification of the intima. As SMCs and macrophages die, cellular debris accumulates to form the core of plaque necrosis, destroying the elasticity of the vessel wall [46, 47]. DO analysis in this study shows that differentially expressed genes related to DNA damage and repair involved in AAD were also involved in atherosclerosis and familial hyperlipidemia, suggesting that these genes may be involved in multiple disease processes inducing AAD and deserve further attention.

Current studies confirmed that the phenotypic transformation, migration and apoptosis of SMCs, and degradation of the extracellular matrix are closely related to the occurrence of AD [48–50]. SMCs maintain vascular stability through their normal contractile capacity and when converted to a synthetic form result in a loss of contractile molecules and an increase in extracellular matrix synthesis [43, 51]. The degree of apoptosis and the mRNA levels of inflammation-related genes were significantly increased in aortic aneurysm and aortic dissection samples [50]. Sirt3 deficiency increases susceptibility to AD development by increasing SMC apoptosis and vascular inflammation [52]. These indicate that the state of SMCs may be related to the occurrence of AAD. Through gene function enrichment analysis, the three biological processes were significantly enriched in AAD, including regulating the cell cycle, response to wounding, and regulating cell adhesion. DNA damage- and repair-related genes may be also involved in AAD by regulating cell death and inflammation, positive regulation of locomotion, and cell death biological processes. This indicates that the genes related to DNA damage and repair may be involved in the occurrence of AAD by regulating SMCs. Additionally, this regulation is not a single factor, but a combination of multiple processes. In the presence of DNA damage in the cells, the tumor suppressor p53 triggers genome-compromised apoptosis, inducing cell cycle arrest or cellular senescence [5, 6, 53]. In abdominal aortic disease, it has been verified that induction of nucleolar stress can trigger a DNA damage response that leads to p53 phosphorylation and cellular senescence in SMCs [40]. This study shows that genes related to DNA damage and repair enriched in the p53 signaling pathway and cellular senescence, so we speculated that these genes involved in AAD might be activating the p53 signaling pathway to regulate SMC senescence or other biological processes' incidence, but this requires more evidence.

Five hub genes involved in the occurrence and development of AAD were screened in this study. The other hub genes were all upregulated in the AAD group, except SLCO2A1. These hub genes have a high diagnostic value for AAD. CDK2, a member of the protein kinase family, encoded protein-formed complexes with cyclins and participates in the progression through the cell cycle [54]. Cell cycle regulation is an important responsibility that allows DNA repair to prevent the wrong passing of genetic material, while if excessive damage persists, cell death processes must be activated to remove damaged cells [55]. CDK plays a crucial role in the initiation of DNA replication, and overactivation of CDK could impair S-phase progression and lead to DNA damage, which made some trouble in research to study mammalian cells [56, 57]. The expression of CDK2 in AD patients increased in bulk RNA sequencing in this study, and scRNA-seq analysis showed that CDK2 was mainly expressed in monocytes and SMCs. The expression of CDK2 increased in monocytes and SMCs in the AAD group, although the difference was not significant.

The protein encoded by the GLRX gene is a member of the glutaredoxin family [58]. GLRX participates in the redox process of various cells by regulating protein S-sulfonylation and is closely related to the occurrence and development of various diseases, such as cardiovascular disease [58, 59], acute lung injury [60], ischemia/reperfusion injury [61], and nonalcoholic fatty liver (NAFL) [62]. A clinical study showed that cardiovascular disease (CVD) patients with high GLRX (>0.622 ng/mL) levels were more likely to experience adverse events [63]. Recent scRNA-seq analysis of aortic aneurysms revealed that GLRX is expressed on SMCs [64]. There is a lack of research on the relationship between GLRX and AD at present. In this study, we found that GLRX was significantly increased in the AAD patients from the bulk RNA sequencing and scRNA-seq, and it was mainly expressed in SMCs and monocytes. This shows that GLRX may be involved in the occurrence of AAD by regulating the redox process of SMCs and monocytes, which needs further experimental verification.

The protein encoded by the NNMT gene is a methyltransferase that catalyzes the formation of N1-methyl nicotinamide from nicotinamide [65, 66]. The NNMT has been found to aggravate inflammatory responses and is associated with the occurrence and development of various diseases and may be a potential therapeutic target for these diseases [66–68]. There is no more evidence for the relationship between NNMT and AAD. This study found that NNMT was upregulated in AAD patients and mainly expressed in SMCs and endothelial cells. The protein encoded by the EIF4A1 gene is involved in cytoplasmic translation initiation and is an important regulator of cancer [69–71]. There is a lack of research to reveal the relationship between EIF4A1 and AAD and AA. This study found that the expression of EIF4A1 was upregulated in the aortic wall tissue of AAD patients. scRNA-seq revealed that EIF4A1 was mainly expressed in SMCs, T cells, and monocytes, which were upregulated in SMCs and monocytes but downregulated in T cells. The differences and functions of EIF4A1 in different cell types need further experimental exploration. The SLCO2A1 gene encodes a prostaglandin transporter. SLCO2A1 is downregulated in the bulk RNA sequencing. scRNA-seq analysis found that SLCO2A1 was mainly expressed in endothelial cells. Currently, there is no evidence revealing its role in the development of AD.

The involvement of inflammation in the development of aneurysms and AD has been demonstrated [72, 73]. Inflammatory cells accumulated in the aortic wall can secrete inflammatory mediators to degrade the extracellular matrix, leading to the weakening of the aortic wall and reducing the ability to resist stress, resulting in ischemia, degeneration, and necrosis of aortic SMCs [74, 75]. The study revealed that there were more immune cells, especially T lymphocytes, in the aortic tissue of patients with ascending AA compared with normal [76]. The T lymphocytes, macrophages, and neutrophils were present in the tissue of AD, with macrophages being the most abundant [77]. Vascular smooth muscle cell-specific EP4 gene knockout (VSMC-EP4-/-) mice treated with angiotensin II (AngII) were more prone to AD and exhibited severe vascular inflammation, macrophage infiltration, and more reactive oxygen species (ROS) [78]. Indomethacin reduces the incidence of AD by inhibiting monocyte transendothelial migration and accumulation of monocytes/macrophages in the aortic wall [79]. In this study, monocytes and M0 macrophages were highly expressed in AAD tissue, which was consistent with previous findings. While naive B cells and *γδ* T cells were lowly expressed in AAD tissues, there are no more data to explain its relationship with AAD. Through correlation analysis, the monocytes were positively correlated with CDK2, EIF4A1, and NNMT and negatively correlated with SLCO2A1. M0 macrophages were positively correlated with NNMT and negatively correlated with SLCO2A1. These indicated that hub genes might participate in the pathology of AAD by regulating the immune cells.

There are still some deficiencies in the study as the data of the study was taken from the public database. Though we verified hub genes in the test dataset, further experiments to verify the hub genes and immune cells are still necessary.

## 5. Conclusions

These results suggested that the DNA damage- and repair-related genes may be involved in the occurrence of AAD by regulating many biological processes, such as inflammatory response and the state of SMCs. At the same time, the four immune cells and five hub genes may be associated with the occurrence of AAD, which may increase the understanding of the AAD mechanism.

## Data Availability

The all datasets used and analyzed in the current research are available from the Gene Expression Omnibus (GEO). The data of this study are also available from the corresponding author on request.

## Supplementary Materials

Supplementary 1 Supplementary Table 1: gene sets that are related to DNA damage and DNA repair were downloaded from the MSigDB database. After screening and deduplication, a total of 1089 genes were included in this analysis.

Supplementary 2 Supplementary Table 2: the data of the gene expression characteristics of 22 kinds of immune cells.

Supplementary 3 Supplementary Table 3: 92 differentially expressed DNA damage- and repair-related genes from the intersection of DEGs of the GSE153434 dataset and DNA damage and repair genes, including 50 upregulated genes and 42 downregulated genes.
