# Supplementary material for: Identification and Analysis of Hub Genes and Immune Cells Associated with the Formation of Acute Aortic Dissection
Source: Comput Math Methods Med. 2023 Feb 8;2023:8072369. doi: 10.1155/2023/8072369 (PMC9936456; doi:10.1155/2023/8072369)
Supplement: Supplementary 4 — Supplementary Figures 1-7. [file 8072369.f4.pdf]

Supplementary Figure 1-7

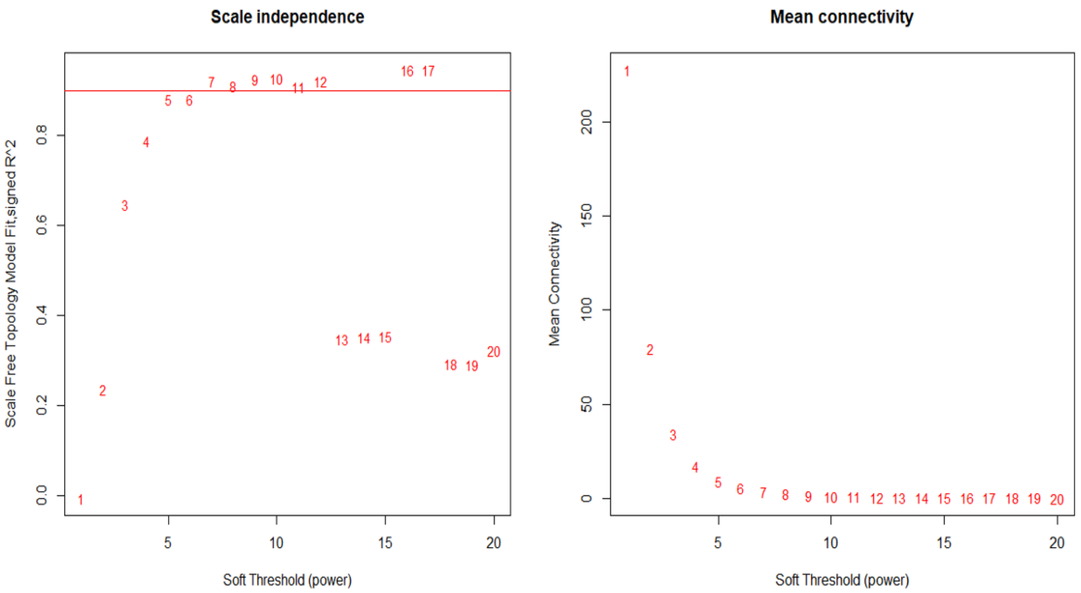

Supplementary Figure 1. Soft threshold analysis suggested gene associations were maximally consistent with the scale-free distribution.

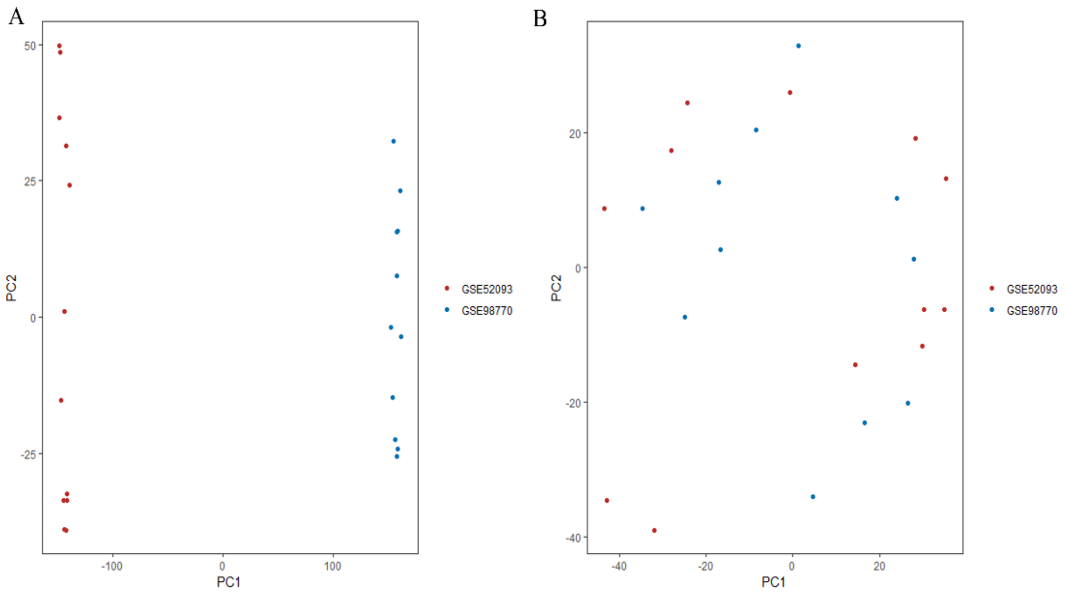

Supplementary Figure 2. Principal component analysis (PCA) before and after merging the GSE52093 and GSE98770.

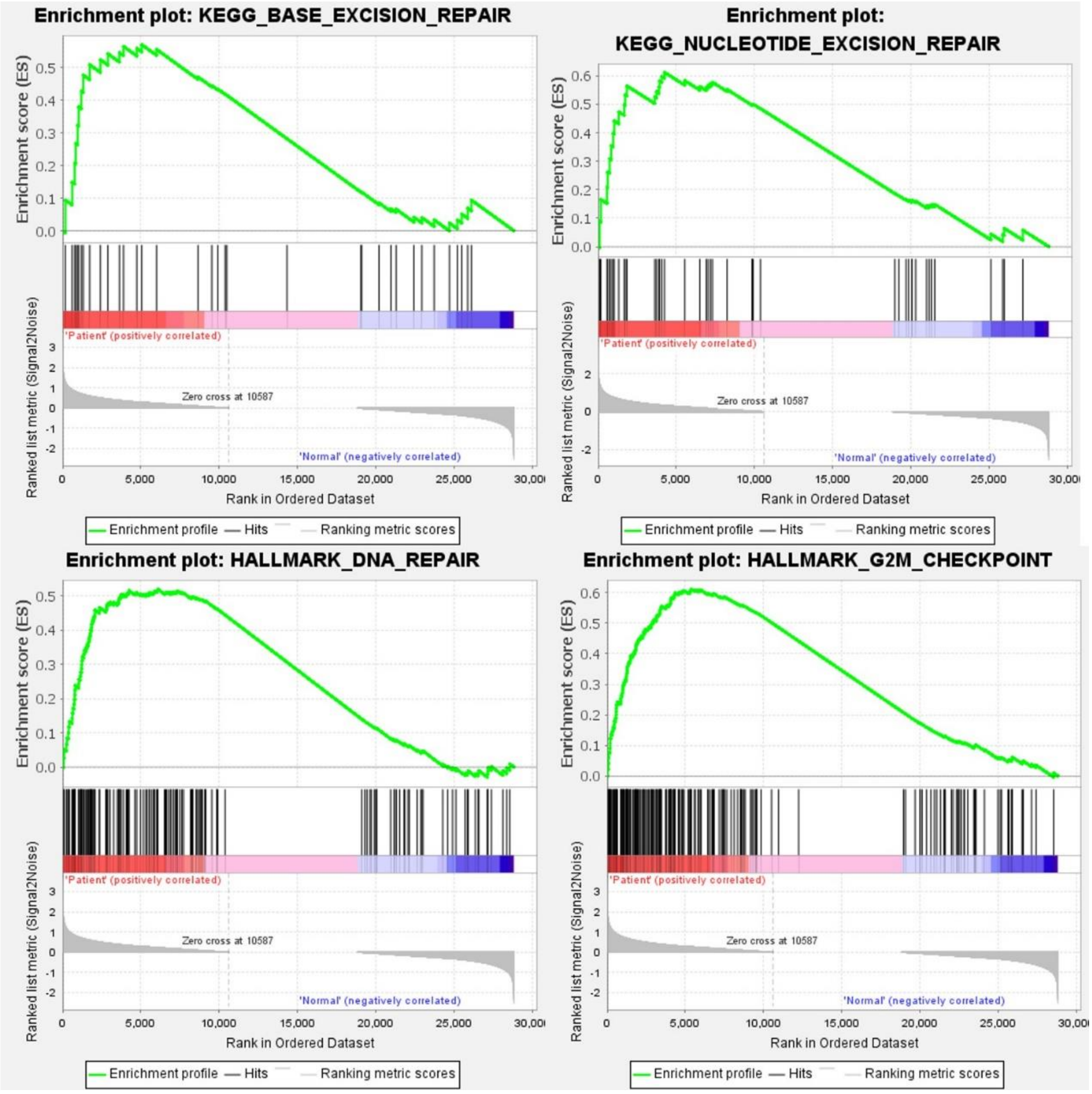

Supplementary Figure 3. The GSEA analysis in GSE153434. Nucleotide excision repair, base excision repair, DNA repair, and G2M checkpoint were significantly enriched in the AD group.

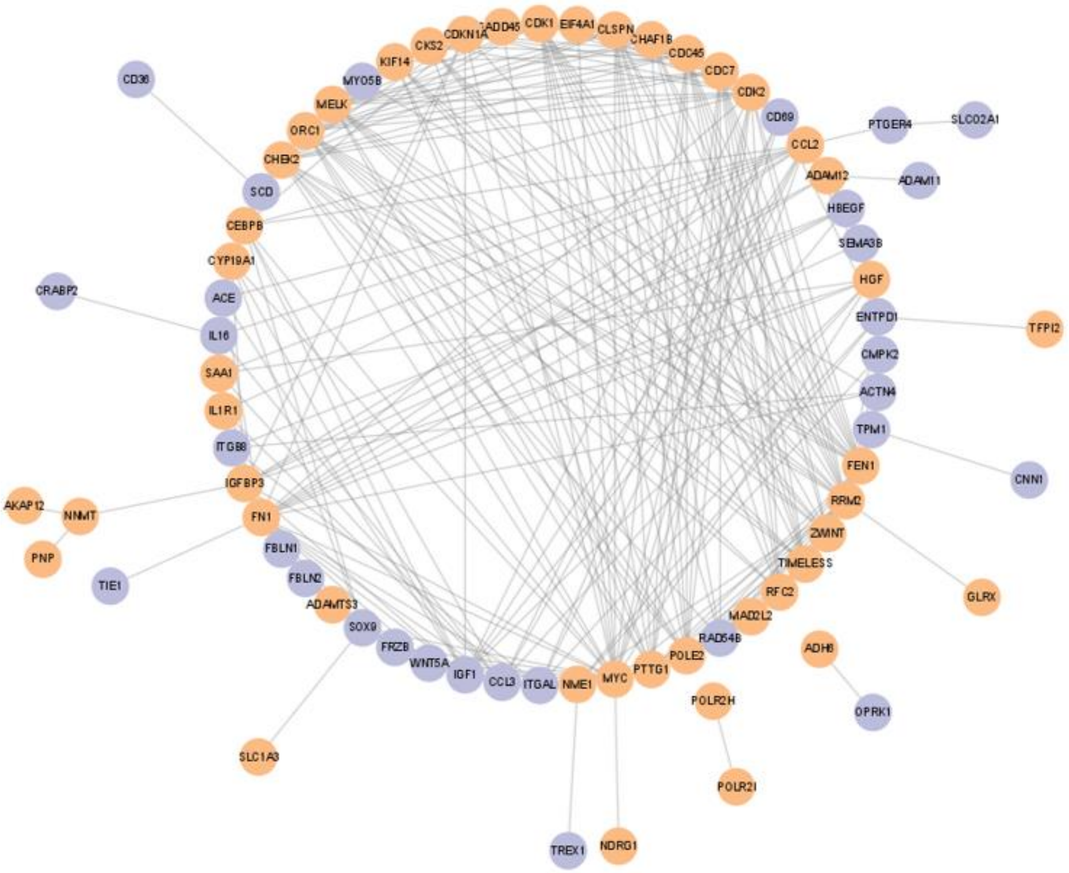

Supplementary Figure 4. The PPI network.

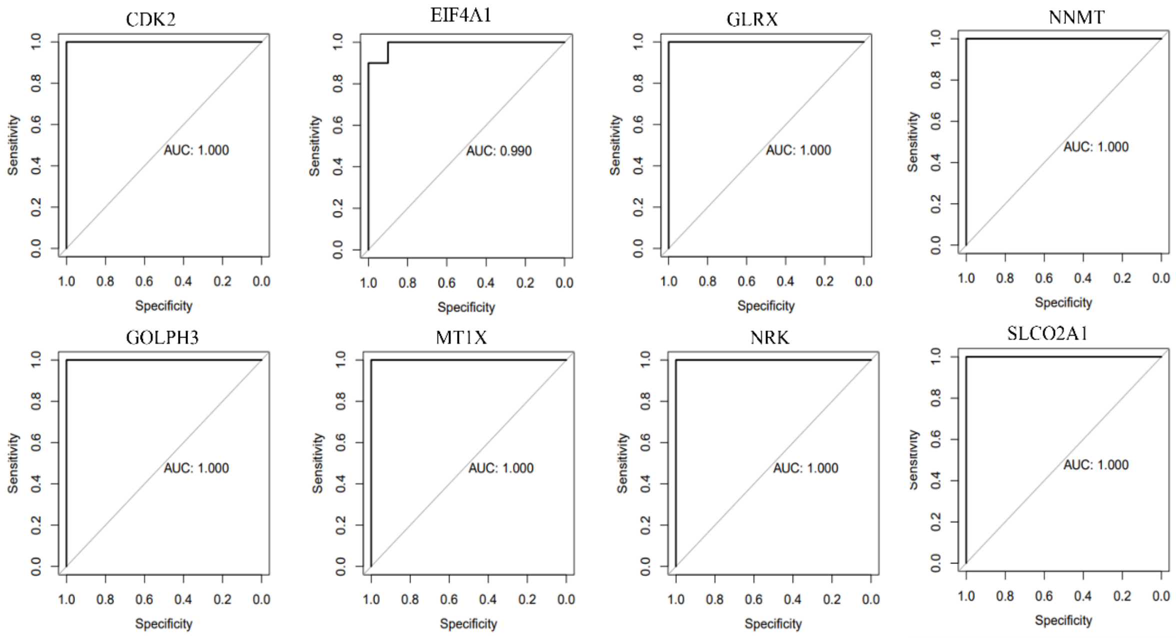

Supplementary Figure 5. ROC curves of hub genes.

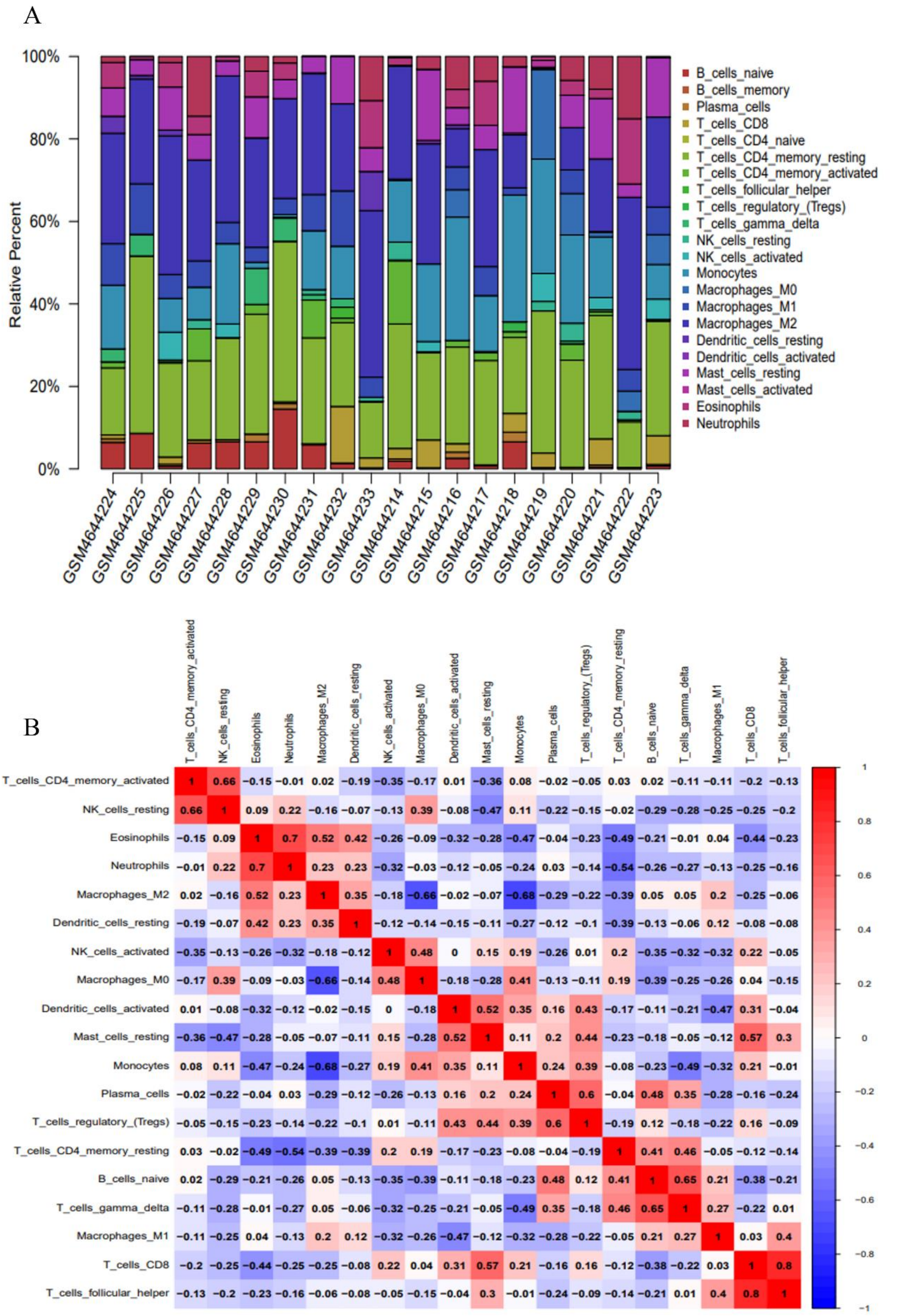

Supplementary Figure 6. Composition of infiltrating immune cells in aortic tissues. (A) The distribution of the immune cell types in each sample. (B) The correlation among immune cell types.

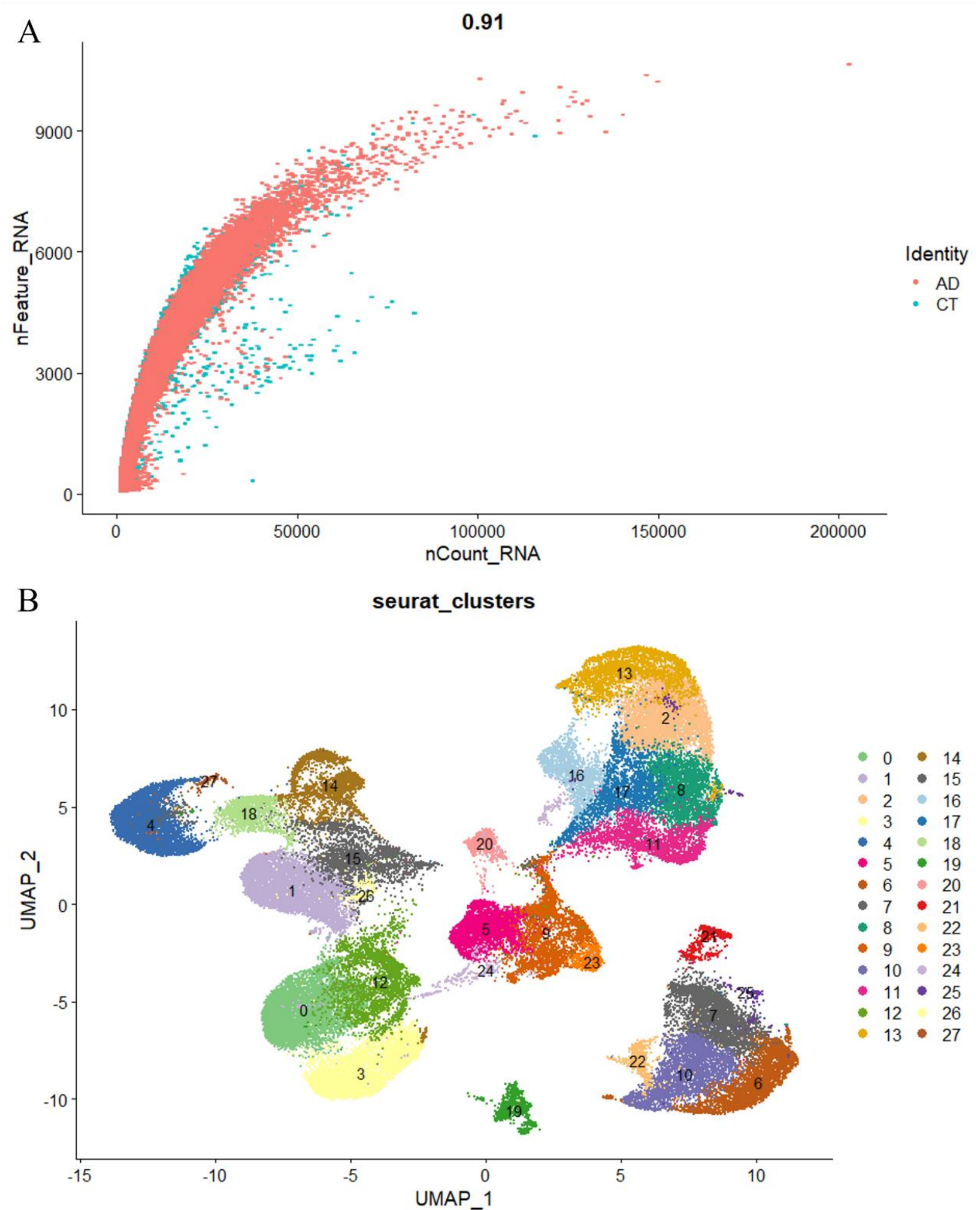

Supplementary Figure 7. (A) The violin plot showed RNA characteristic number (nFeature RNA) and absolute UMI count (nCount RNA) after low-quality cells were removed. (B) The cell clusters.
